# Efficacy and Safety of Sorafenib Therapy on Metastatic Renal Cell Carcinoma in Korean Patients: Results from a Retrospective Multicenter Study

**DOI:** 10.1371/journal.pone.0135165

**Published:** 2015-08-26

**Authors:** Sung Han Kim, Sohee Kim, Byung-Ho Nam, Sang Eun Lee, Choung Soo Kim, Ill Young Seo, Tae Nam Kim, Sung-Hoo Hong, Tae Gyun Kwon, Seong Il Seo, Kwan Joong Joo, Kanghyon Song, Cheol Kwak, Jinsoo Chung

**Affiliations:** 1 Departments of Urology, Center for Prostate Cancer, National Cancer Center, Goyang, Korea; 2 Biometric Research Branch, Center for Prostate Cancer, National Cancer Center, Goyang, Korea; 3 Seoul National University Bundang Hospital, SeongNam, Korea; 4 Asan Medical Center, Seoul, Korea; 5 Samsung Medical Center, Sungkyunkwan University School of Medicine, Seoul, Korea; 6 Pusan National University Hospital, Busan, Korea; 7 Seoul St. Mary’s Hospital, Seoul, Korea; 8 School of Medicine, Kyungpook National University, Daegu, Korea; 9 Wonkwang University School of Medicine and Hospital, Iksan, Korea; 10 Kangbuk Samsung Hospital, Sungkyunkwan University School of Medicine, Seoul, Korea; 11 Korea Cancer Center Hospital, Seoul, Korea; 12 Seoul National University Hospital, Seoul, Korea; Seoul National University, REPUBLIC OF KOREA

## Abstract

**Objective:**

To evaluate the efficacy and safety of sorafenib for Korean patients with metastatic renal cell carcinoma (mRCC).

**Methods:**

A total of 177 mRCC patients using sorafenib as first- (N = 116), second- (N = 43), and third-line (N = 18) therapies were enrolled from 11 Korean centers between 2006 and 2012. The patient characteristics, therapy duration, tumor response, disease control rate, and tolerability were assessed at baseline and at routine follow-ups, and the progression-free survival (PFS) and overall survival (OS) times and rates were analyzed.

**Results:**

Among all patients, 18 (10.2%) stopped sorafenib treatment for a median of 1.7 weeks, including 15 (8.5%) who discontinued the drug, while 40 (22.6%) and 12 (6.8%) patients required dose reductions and drug interruptions, respectively. Severe adverse events (AEs) or poor compliance was observed in 64 (36.2%) patients, with 118 (7.4%) ≥grade 3 AEs. During the treatment, one myocardial infarction was observed. The number of ≥grade 3 AEs in the first-line sorafenib group was 71 (6.8% of the total 1048 AEs). During a median follow-up of 17.2 months, the radiologically confirmed best objective response rate, disease control rate, median PFS, and median OS were 22.0%, 53.0%, 6.4 months (95% confidence interval [CI], 5.2–8.9), and 32.6 months (95% CI, 27.3–63.8) for the total 177 sorafenib-treated patients, respectively, and 23.2%, 56.0%, 7.4 months (95% CI, 5.5–10.5), and not reached yet (95% CI, 1.0–31.1) for the first-line sorafenib group, respectively.

**Conclusions:**

Sorafenib produced tolerable safety, with a ≥grade 3 AE rate of 7.4% and an acceptable disease control rate (53.0%) in Korean mRCC patients.

## Introduction

Until the early 2000s, which saw the advent of targeted therapy (TT), metastatic renal cell carcinoma (mRCC) was considered a dismal disease, owing to its high resistance to chemotherapy and poor responses (<20%) to cytokine therapy, the first-line systemic therapy for mRCC patients at that time [1]. Moreover, many patients were unable to receive cytokine therapy, particularly interleukin-2, due to toxicity, resulting in 5-year overall survival (OS) rates of only 10–22% [2].

After the introduction of several new agents, the standard first-line treatment for advanced RCC has shifted to TT. Among the TTs, sorafenib (Nexavar, Bayer Healthcare Pharmaceuticals, Wayne, NJ, USA, and Onyx Pharmaceuticals, South San Francisco, CA, USA) is a multi-targeted tyrosine kinase inhibitor against the vascular endothelial growth factor and platelet-derived growth factor receptors, Fms-like tyrosine kinase 3, ret proto-oncogene, proto-oncogene c-kit, and the raf serine/threonine kinases B-RAF and C-RAF [3]. The efficacy of sorafenib in RCC has been confirmed in many trials from different countries, and sorafenib is now available worldwide for the treatment of advanced RCC [3–10].

In Korea, 3 years after the US Food and Drug Administration approved sorafenib in December 2005 for RCC, the Ministry of Food and Drug Safety approved it as a first-line treatment for advanced RCC. Until then, immunochemotherapy had been widely used, with disappointing outcomes, and no standard treatment existed for immunochemotherapy-refractory patients.

Nowadays, with the satisfied efficacy and safety outcomes of first-line sorafenib, several comparative studies have reported the efficacy of sorafenib as second- and third-line TT in failed mRCC patients to prior TTs [11–14]. However, few studies have examined its effectiveness and safety in Asian ethnic patients, especially Koreans [8, 13, 15, 16], and there is currently no multicenter study investigating the efficacy of sorafenib in Korea.

Therefore, this study, comprising mRCC clinical data from 11 academic Korean centers, aimed to evaluate the safety and efficacy, including the therapeutic responses, of sorafenib as first-, second-, and third-line treatments.

## Material and Methods

Clinically diagnosed mRCC patients (N = 184) from 11 Korean academic hospitals, treated with sorafenib as TT with/without prior systemic therapy between 2006 and 2012, were retrospectively reviewed. After excluding patients aged <18 years and with a life expectancy <3 months, 177 patients were enrolled, including 116 (65.5%), 43 (24.3%), and 18 (10.2%) patients treated with sorafenib as first-, second-, and third-line TT, respectively. RCC was pathologically confirmed from the primary or metastatic site(s) by nephrectomy, metastasectomy, or tumor biopsies, and the tumors were staged according to the 2009 American Joint Committee on Cancer classification as stage IV.

Sorafenib was administered based on the treatment recommendation by the US Food and Drug Administration for mRCC, starting with 400 mg orally twice daily, at 12-hour intervals, on a continuous basis [3, 17]. Dose modification to 400 mg once daily was permitted based on the clinician's judgment according to the tumor response and adverse events (AEs; grade 3 or 4 toxicity), as defined by the Common Terminology Criteria for Adverse Events v.3.0 [18]. The treatment continued until disease progression or treatment intolerance developed. The tumor response was measured for 4–12 weeks after the treatment initiation using the Response Evaluation Criteria in Solid Tumors v.1.1 criteria [19].

Baseline demographic and clinicopathological data were collected (Table 1); the pre- and post-treatment evaluations consisted of complete history taking and physical examination, complete blood count, liver and renal function tests, chest computed tomography (CT), abdominal and pelvis CT or magnetic resonance imaging, and total body bone scan. Fluorodeoxyglucose-positron emission tomography or positron emission tomography/CT scan were optional. During the treatment, all patients were evaluated according to the institutional protocol by their attending urologists, and follow-up visits were regularly conducted after the treatment termination until death.

**Table 1:** Baseline characteristics (N = 177).

Parameter	N (%) or median (range)
Gender (male/female)	136/41
Age (years)	62.0±10.9
Follow-up duration (months)	17.2 (0.2–63.8)
Treatment duration (weeks)	20 (1–216)
Body mass index (kg/m^2^)	23.3 (14.5–37.2)
Comorbidity	
Cerebrovascular accident	5 (2.8)
Angina	2 (1.1)
Myocardial infarction	4 (2.3)
Thrombosis/embolism	1 (0.6)
Deep vein thrombosis	1 (0.6)
Hypercholesterolemia	3 (1.7)
Hyperlipidemia	2 (1.1)
Body surface area (m^2^) ≤1.7	74 (46.0)
>1.7	87 (54.0)
Unknown	16
Karnofsky performance score >80	107 (78.7)
50–80	22 (16.2)
<50	7 (5.1)
Unknown	41
MSKCC risk criteria, Favorable	49 (35.0)
Intermediate	82 (58.6)
Poor	9 (6.4)
Unknown	37
Heng risk criteria, Favorable	39 (28.7)
Intermediate	78 (57.3)
Poor	19 (14.0)
Unknown	41
Prior surgical therapy	
Nephrectomy [radical/partial/embolization]	150[135/5/10] (66.9)
Metastasectomy	27 (23.1)
Prior systemic therapy	56 (31.6)
Immunotherapy	33 (18.6)
Chemotherapy	4 (2.3)
Targeted therapy (sunitinib)	19 (10.7)
Radiation therapy	18 (10.2)
Primary renal tumor in situ	32 (18.1)
Number of metastatic sites (organs)	3 (1–5)
1 organ	94 (57.0)
2 organs	44 (26.7)
3 organs	19 (11.5)
≥4 organs	8 (4.8)
Unknown	12
Metastatic sites	
Brain	42 (23.7)
Bone	38 (21.5)
Liver	17 (9.6)
Lung	124 (70.1)
Lymph nodes	34 (19.2)
Pancreas	8 (4.5)
Kidney, contralateral	7 (4.0)
Other	30 (16.9)
Characteristics of primary renal tumor	8 (1–117)
Size of primary tumor (cm)	
Collecting system invasion	28 (15.8)
Capsule invasion	36 (20.3)
Lymphovascular invasion	34 (19.2)
Tumor necrosis	46 (26.0)
TNM stage T1	25 (14.1)
T2	35 (19.8)
T3	74 (41.8)
T4	8 (4.5)
Tx	35 (19.8)
N1	27 (15.3)
M1	131 (74.0)
Fuhrman grade 1	5 (3.4)
2	39 (26.2)
3	69 (46.2)
4	36 (24.2)
Unknown	28
Histology, Clear cell, pure	159 (98.1)
Non-clear cell	3 (1.9)
Unknown	15
Best overall response (CR+PR+SD)	94 (53.1)
Complete remission (CR)	6 (3.4)
Partial response (PR)	33 (18.6)
Stable disease (SD)	55 (31.1)
Progressive disease	83 (46.9)
Progression-free survival (median, months)	6.4 (5.2–8.9)
Overall survival (median, months)	32.6 (27.3–63.8)
Survival	89 (74.8)
Cancer-specific death	19 (16.0)

MSKCC, Memorial Sloan-Kettering Cancer Center; TNM, tumor-node-metastasis.

The disease control rate was defined as the proportion of patients who achieved stable disease (SD), partial response (PR), or complete response (CR). Progression-free survival (PFS) was defined from the date of sorafenib initiation until documented radiologically confirmed disease progression or death. OS was defined from the date of sorafenib initiation until all-cause death. Continuous variables are summarized as medians and ranges, and categorical variables as proportions. Kaplan-Meier analyses were used for estimating PFS and OS. All statistical analyses were performed using STATA software (version 13.1, STATA Inc., TX, USA) by two medical statisticians (Nam BH, PhD and Kim SH, PhD).

### Ethical statement

All study protocols were conducted according to the ethical guidelines of the World Medical Association Declaration of Helsinki-Ethical Principles for Medical Research Involving Human Subjects [20]. This retrospective study was approved by the Institutional Review Board of the Research Institute and Hospital of National Cancer Center, Goyang, Korea (IRB No. NCCNCS 11–439) and the Institutional Review Boards of the 11 participating hospitals, including Seoul National University Bundang Hospital, SeongNam; Asan Medical Center, Seoul; Samsung Medical Center, Sungkyunkwan University School of Medicine, Seoul; Pusan National University Hospital, Busan; Seoul St. Mary’s Hospital, Seoul; School of Medicine, Kyungpook National University, Daegu; Wonkwang University School of Medicine and Hospital; Kangbuk Samsung Hospital, Sungkyunkwan University School of Medicine, Seoul; Korea Cancer Center Hospital, Seoul; and Seoul National University Hospital, Seoul. The Institutional Review Boards of all 11 participating hospitals waived the need for written informed consent from the participants. All patient records/information were anonymized and de-identified prior to analysis in this study.

## Results

**Fig 1:**
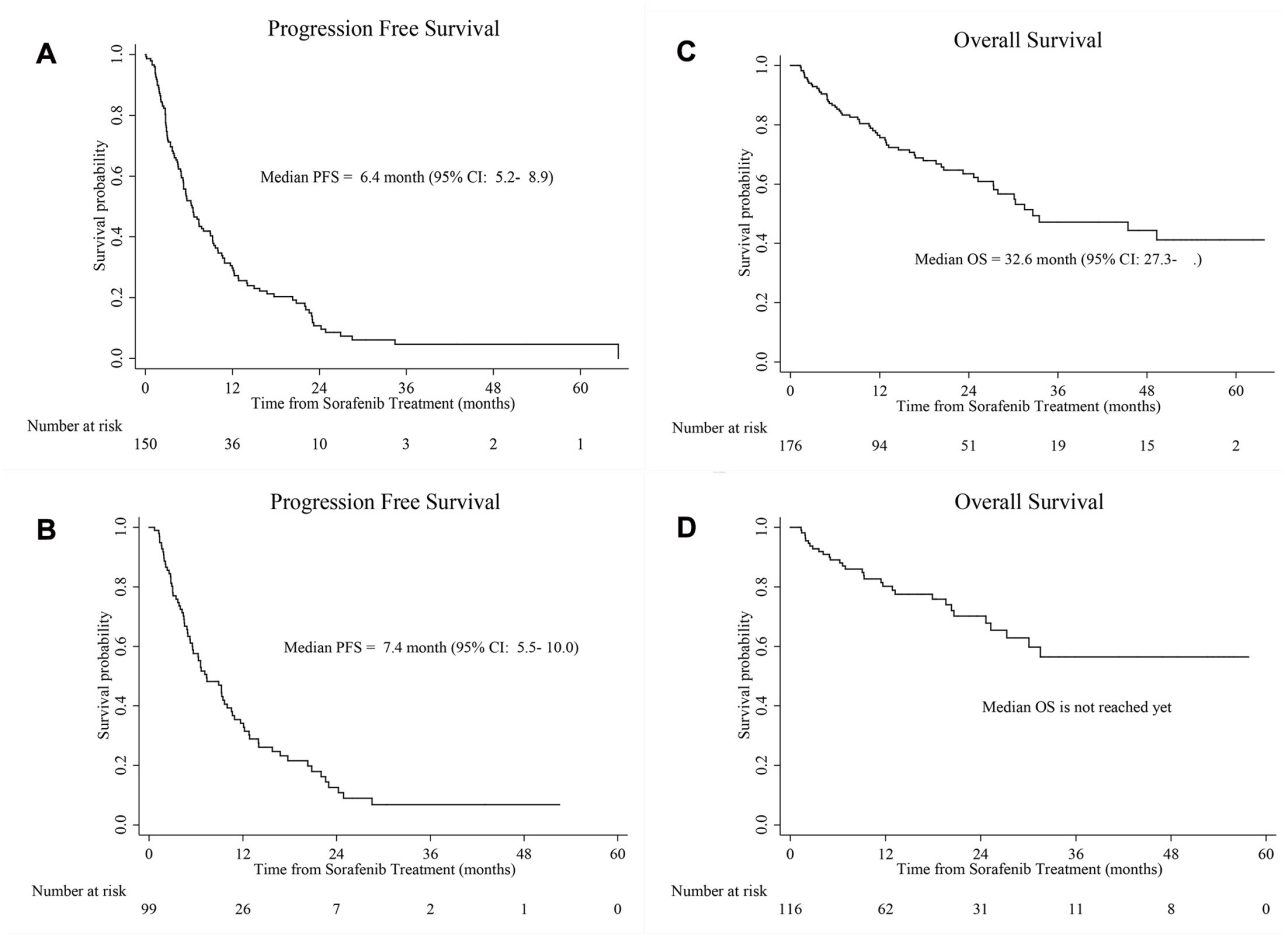
Progression-free (PFS) and overall survivals (OS) for (A, C) all 177 patients and (B, D) 116 patients treated with first-line sorafenib, respectively. CI, confidence interval.

During a median follow-up of 17.2 months, the radiologically confirmed best objective response rate (ORR; CR+PR), disease-control rate (DCR; CR+PR+SD), and median PFS and OS times were 22.0%, 53.1%, and 6.4 and 32.6 months, respectively (Table 1). The corresponding values in the 116 first-line sorafenib patients were 23.2%, 56.0%, 7.4 months, and unreached (>31.1 months), respectively. For the 43 second-line and 18 third/fourth-line patients, the DCRs were 46.5% (2.3+16.3+27.9%) and 38.9% (0+16.7+22.2%), respectively (Table 2, Fig 1).

**Table 2:** Description of ≥ graded 3 adverse events during sorafenib treatment (N,%) (See also S1 Table, for more details).

Adverse event	First-line (N = 116)	Overall (N = 177)
**Constitutional symptoms**		
Fatigue		2 (1.1)
Weight loss		1 (0.6)
**Gastrointestinal**		
Diarrhea	1 (0.8)	4 (2.3)
Vomiting	1 (0.8)	1 (0.6)
Anorexia	3 (2.5)	4 (2.3)
Heartburn	1 (0.8)	1 (0.6)
Dental problems	1 (0.8)	1 (0.6)
Mucositis/stomatitis	1 (0.8)	2 (1.1)
**Dermatological**		
Rash/desquamation	3 (2.5)	4 (2.3)
Rash: hand-foot skin reaction	11 (9.2)	18(10.2)
Dermal change	2 (1.7)	2 (1.1)
Alopecia	1 (0.8)	2 (1.1)
**Cardiac**		
Hypertension	1 (0.9)	7 (4.0)
Cardiac ischemia/infarction	1 (0.9)	1 (0.6)
**Pulmonary/upper respiratory**		
Dyspnea	1 (0.8)	1 (0.6)
Voice changes	1 (0.8)	2 (1.1)
**Other**		
Pain	2 (1.7)	3 (1.7)
**Hematologic**		
Anemia	8 (6.9)	10 (5.6)
Lymphopenia	3 (2.6)	3 (1.7)
Neutrophil/granulocyte (ANC/AGC)	2 (1.7)	2 (1.1)
**Non-hematologic**		
Elevated AST	2 (1.7)	2 (1.1)
Elevated ALT	3 (2.6)	4 (2.3)
Hyperbilirubinemia	1 (0.9)	1 (0.6)
Hypocalcemia	1 (0.9)	2 (1.1)
Elevated creatinine	3 (2.6)	3 (1.7)
Hyperglycemia		3 (1.7)
Elevated amylase	2 (1.7)	5 (2.8)
Elevated lipase	4 (3.4)	8 (4.5)
Hypophosphatemia	9 (7.8)	14 (7.9)
Hyperkalemia	1 (0.9)	2 (1.1)
Hyponatremia	1 (0.8)	2 (1.1)
Hyperuricemia		1 (0.6)
**Total**	**71**	**118**

ANC, absolute neutrophil count; AGC, absolute granulocyte count; AST, aspartate transaminase; ALT, alanine transaminase.

In terms of the safety profile, 1595 AEs occurred among 112 (63.3%) patients during the median 20-week treatment duration; of these, 118 (7.4%) AEs ≥grade 3 in 64 (36.2%) patients, including one myocardial infarction, negatively affected the treatment tolerability (Table 2). Among these 64 patients, 40 (62.5%) received first-line sorafenib, with a ≥grade 3 AE rate of 6.8% observed (71/1048 AEs). The AEs ≥grade 3 included hand-foot syndrome (10.2%), anemia (5.6%), hypertension (4.0%), and serum lipase elevation (4.5%), with the most common AEs to cause discontinuation, reduction, or interruption of sorafenib being hand-foot skin reaction (68.9%), general fatigue (55.1%), anorexia (44.8%), nausea (44.0%), anemia (42.2%), and rash (42.2%).

Among all 177 patients, 15 (8.5%), 40 (22.6%), and 12 (6.8%) patients required drug discontinuation, dose reduction, and drug interruption, respectively, for a median of 1.7 weeks due to severe AE or poor compliance, resulting in 18 (10.2%) patients stopping treatment (data not shown). For the first-line sorafenib group, during a median of 5.6 (range, 1.2–41.1) months of treatment, a daily dose of 800 mg of sorafenib was initiated in 116 patients, whereas 28 (24.1%) patients required dose reduction and 11 (9.5%) interrupted treatment due to severe AEs, with a median maximum duration of 1.7 (range, 0.7–18.2) months (Table 3). However, only 9 (7.8%) patients finally discontinued sorafenib due to severe AEs.

**Table 3:** Therapeutic responses to sorafenib according to the treatment line.

Category	Number (percentage) or median (min-max)		
	First-line (N = 116, 65.5%)	Second-line (N = 43, 24.3%)	Third-line (N = 18, 10.2%)
Best overall response (CR+PR+SD)	65 (56.0)	20 (46.5)	7 (38.9)
Complete remission (CR)	4 (3.4)	1 (2.3)	0 (0.0)
Partial response (PR)	23 (19.8)	7 (16.3)	3 (16.7)
Stable disease (SD)	38 (32.8)	12 (27.9)	4 (22.2)
Progressive disease	38 (32.8)	18 (41.9)	4 (22.2)
Not evaluated	13 (11.2)	5 (11.6)	7 (38.9)
Progression-free survival (months)	7.4 (5.5–10.5)	5.2 (2.9–7.4)	2.9 (1.4–8.0)
One-year progression-free survival	34.1 (24.2–44.3)	22.6 (10.7–37.3)	12.5 (0.7–42.3)
Overall survival (months)	NR (1.0–31.1)	27.4 (12.7–33.5)	16.0 (4.9-NR)
One-year overall survival	80.2 (70.8–86.8)	69.2 (51.9–81.3)	75.0 (31.5–93.1)
Drug discontinuation	69 (59.5)	27 (62.8)	5 (27.8)
Adverse events	9 (13.0)	1 (3.7)	2 (40.0)
Poor compliance	3 (4.3)	0 (0.0)	0 (0.0)
Loss of follow-up	5 (7.2)	3 (11.1)	0 (0.0)
Disease progression	43 (62.3)	19 (70.4)	3 (60.0)
Death	6 (8.7)	1 (3.7)	0 (0.0)
Other	3 (4.3)	3 (11.1)	0 (0.0)
Dose reduction	41 (35.3)	4 (9.3)	1 (5.6)
Adverse event	28 (68.3)	4 (100.0)	0 (0.0)
Poor compliance	7 (17.1)	0 (0.0)	1 (100.0)
Other	6 (14.6)	0 (0.0)	0 (0.0)
Dose interruption	16 (13.8)	3 (7.0)	1 (5.6)
Adverse event	11 (68.8)	1 (33.3)	0 (0.0)
Other	5 (31.3)	2 (66.7)	1 (100.0)
Duration of dose interruption (weeks)	1.4 (0.7–78)	2.1 (1.6–3)	2 (2–2)

NR, not reached yet.

## Discussion

While the efficacy and safety of sorafenib for mRCC have been confirmed by an international collaborative study mostly comprising Western patients, which reported significant improvements in the PFS and OS and an acceptable tolerability with multiple AEs [21], the efficacy and safety of sorafenib in Asian populations remain unclear. Further, no Korean study on sorafenib in mRCC patients with/without prior systemic therapy currently exists, with the exception of some small single-center studies [22, 23]. The current retrospective multicenter study comprising 11 Korean academic hospitals revealed the efficacy and safety of sorafenib treatments as first-, second-, and third-line therapy in a representative set of Korean patients with mRCC.

**Fig 2:**
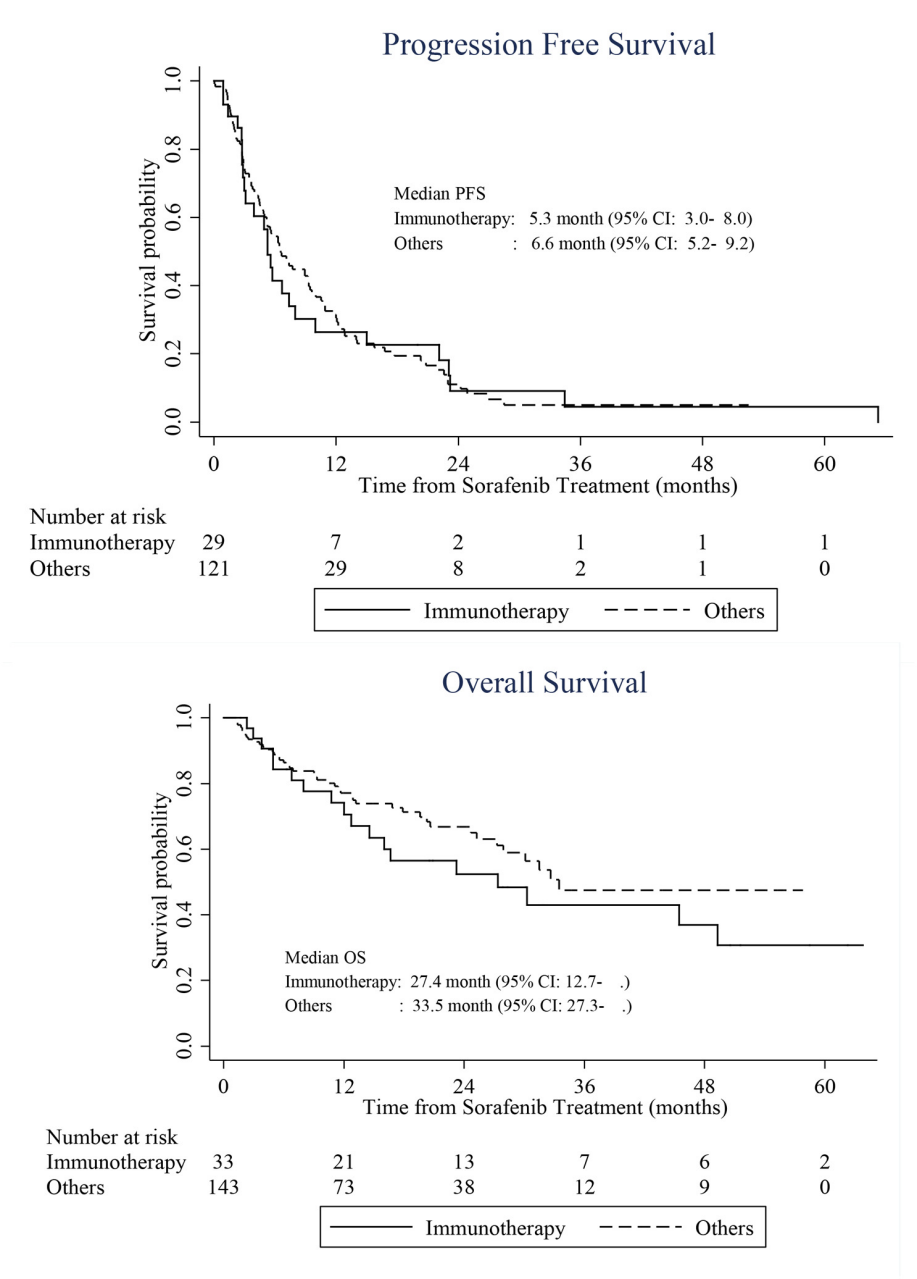
Comparison of progression-free (PFS) and overall survivals (OS) after sorafenib treatment between immunotherapy-refractory (N = 33) and non-immunotherapy patients (N = 144). CI, confidence interval.

In this study, the ORR, DCR, PFS, and OS were 22.0%, 51.1%, and 6.4 and 32.6 months, respectively (Table 1). Comparison of the sorafenib responses between patients with prior immunotherapy or other systemic therapy, and those without prior systemic therapy showed that the immunotherapy group tended to have slightly worse PFS (5.3 vs. 6.6 months, p = 0.863) and OS (27.4 vs. 33.5 months, p = 0.230), although these differences were not statistically significant (Fig 2).

Moreover, compared to previous Western [3, 4, 6], Asia-Pacific [15], and Japanese and Chinese studies [24, 25], our study showed similar or better PFS and OS, and better ORR (22.0%) than the Western studies, and similar or lower response rates and prognoses than the adjacent Asia-Pacific countries (Table 4). However, the DCR of this study (51.1%) was worse than that in both the previous Western and Asia-Pacific trials, likely owing to the fact that all non-evaluated patients in this study were presumed progressed when the overall best response of sorafenib was assessed. However, other Japanese studies showed similar efficacies of sorafenib to ours [25–28].

**Table 4:** Comparison of studies using sorafenib in advanced renal cell carcinoma from Korea and other nations, including Japan, China, and Western countries.

Study name/location (reference number)	Prior cytokine use (%)	Objective response rate (%)	Disease control rate (%)	Median PFS (months)	Median OS (months)
This study	18.6	22.0	53.1	6.4	32.6
Korea (23, 41)	12–29	24–30	60–71	8.6	25.7
TARGET (3)	100	10.2	84.0	5.5 (24weeks)	17.8 (78weeks)
EU-ARCCS (4)	67	4	85	6.6 (28.3weeks)	NE
NA-ARCCs (6)	50	4	84	5.6 (24weeks)	11.7 (50weeks)
PREDICT (15)	41.0	23.3	70.3	7.3	NE
AXIS II (34)	35	3.4–9.4	58.6–63.8	4.7–4.9	NE
China (9, 10)	39.7–45.5	16.7–24.5	80.0–87.8	14–15 (40weeks)	16.0–16.1 (69 week)
China (8, 10)	82.2–91.9	21.0–36.6	84.2–88.1	9.6–9.7(41-45weeks)	NE
Japan (8)	50–60	19.4–21.8	52.0–73.6	7.3–12.2	11.9–32.5
Western trials (15, 32, 33)	50–100	4–10.2	84–85	5.5–6.6 (24–28.3 weeks)	11.7–17.8 (50–76 weeks)

OS, overall survival; PFS, progression-free survival; NE, not evaluated.

The better efficacy and clinical outcomes of sorafenib in Asian compared to Western patients with mRCC has already been reported [7, 10]. The potential reasons for these differential outcomes include ethnic differences, including differential expressions of tumor markers and molecular features, as has been previously demonstrated in other malignancies [10]. Furthermore, the patient characteristics differed between each study, with our study having a relatively small portion of immunotherapy-refractory patients (18.6%) and more patients with advanced disease (median, three metastatic sites).

Another interesting point of this study was that the failed immunotherapy patients (N = 33) had worse PFS (5.3 months) and OS (27.4 months) than other patients (N = 144; PFS, 6.6 months; OS, 33.5 months) (Fig 2). This is likely because 80.6% (N = 116) of the non–immunotherapeutic patients were treatment-naïve mRCC patients who received sunitinib and sorafenib as first- or second-line therapy, thereby resulting in better clinical outcomes. Similarly, other comparative studies also reported that the sorafenib efficacy in advanced RCC with prior cytokine therapy was similar or tended to be slightly decreased compared to advanced RCC without prior cytokine therapy but with other TTs [4, 7, 10, 29, 30], and this is why sorafenib is now used for first- and second-line therapy for advanced RCC rather than cytokine therapy.

As for the outcomes in the first-line setting in mRCC patients (N = 116), the ORR, DCR, PFS, and OS were 23.2%, 56.0%, 7.4 months, and unavailable (censored at 30.1 months), respectively (Table 2). Escudier et al., in their randomized phase II trial comparing first-line sorafenib to interferon-alpha therapy, reported median PFSs of 5.6 and 5.7 months, respectively [31]. Further, in the PREDICT study, first-line sorafenib was associated with a median PFS of 7.6 months, without available OS data [15], similar to in our study.

Western studies have reported ORRs, PFSs, and OSs of 4–10.2%, 5–6 months, and 11.7–17.8 months, respectively [3, 4, 6, 31], and another recent sorafenib comparison study with the third-generation tyrosine kinase inhibitor tivozanib showed PFS and OS of 9.1 and 29.3 months, respectively in the sorafenib group, which were better than the results of our and previous studies [32]. This difference likely results from differences in the enrolled patients. The tivozanib trial comprised mRCC patients from East-Europe with performance statuses of 1 and 2, who all underwent nephrectomy, had no prior immunochemotherapy, and had confirmed clear cell pathology.

Compared to other studies from Asia, with similar ethnic populations as Korea, the Japanese experiences of sorafenib as first-line therapy were similar to ours, in which the DCR reached 46–59.9%, ORR 21.8–22%, and PFS 7.9–9 months [25, 26]. However, in Chinese studies, better or similar responses were reported, with radiologically confirmed DCRs of 21.0–24.5%, DCR of 87.8% (CR+PR+SD>4 months), median PFS of 60 weeks, and unreached median OS until 76 weeks’ follow-up [9, 10].

The use of sorafenib as second- (N = 43) and third-line (N = 12) therapies for mRCC patients with prior systemic history was associated with ORR, DCR, PFS, and OS of 18.6%, 44.5%, 5.2 months, and 27.4 months, and 16.7%, 50.0% (without CR), 2.9 months, and 16.0 months, respectively (Table 2). The previous second- and third-line sorafenib studies showed ad ORR of 10.1%, with PFS and OS of 3.9–4.8 and 16.6–19.2 months, respectively, for second-line sorafenib, and a PR rate of 4% and PFS of 3.6 months for third-line TT [13, 33].

Among the 43 patients treated with second-line sorafenib, those undergoing prior immunotherapy (N = 23) had better PFS and OS than patients with prior sunitinib history (N = 17; 5.6 [0.6–3.1] and 27.4 months for immunotherapy, and 3.5 [1.5–7.7] and 12.9 months for sunitinib therapy, respectively; data not shown). These results were similar to those of the AXIS phase 3 trial, with median PFSs of 6.5–6.6 and 2.8–3.4 months for the cytokine and sunitinib groups, respectively [34].

Compared with our study, it might be concluded that similar best overall responses and prognoses, including PFS and OS, were achieved in Asian mRCC patients treated with sorafenib in the first-, second-, and third-line settings, whereas the efficacy was poorer in Western patients [24–26], suggesting that ethnicity-related differences in the genetic profiles, tumor marker expressions, and molecular features, as well as differences in body composition, are important factors for therapeutic efficacy [35, 36]. Accordingly, previous studies on mRCC and other malignancies have indicated that Asian mRCC patients have better clinical outcomes compared with Western patients [7, 10]. Furthermore, molecular targeting studies have also shown different efficacies among patients of different ethnicities and with different cancer types [7]. Whether these differences are induced by genetic and molecular factors remains unknown; however, it is reasonable to postulate that genetic differences in the tumor cells play important roles in determining the differences in the disease phenotype.

As mentioned above, another potential explanation for the different efficacies and safety profiles observed between different ethnicities might be differences in the body composition, such as the body surface area. These differences, together with genetic factors might explain the longer median survival time of Japanese mRCC patients compared to North American or European patients in the cytokine era [35, 37]. Differences in the sorafenib concentrated volume and maintenance doses, as assessed by expression of tumor markers and molecular features, in patients from different ethnic groups with different body sizes have been well documented for many malignancies from different organs, and numerous studies have shown the importance of body shape as a risk factor for the therapeutic efficacy of cancer therapy and prognosis [35, 38].

Van der Veld et al. showed that severe AEs (causing dose reductions or permanent discontinuation) highly correlated with low body surface area [35]. Asians have significantly smaller body volume than Western populations, and some Japanese studies have suggested an optimal dose of 600 mg for Japanese patients, with maintenance of at least half the dose for the first month of therapy [26, 34]. This might also be the reason for the higher rates of AEs and therapeutic interruption in Asian studies, including this study, compared to in Western studies [35, 36, 39].

In this study, in the first-line sorafenib setting, 28 (24.1%) patients required dose reduction, 11 (9.5%) required interruption, and 9 (7.8%) required treatment discontinuation due to AEs, which were higher than the rates in Western studies such as the TARGET and European SHARP studies (28%) [3, 40, 41] (Table 3). Asian (particularly Korean, Japanese, and Chinese) patients are more prone to certain AEs, especially hand-foot skin reaction (67.2% in this study and 55–88.1% in previous Japanese and Chinese reports) [9, 24] compared to Western patients (10–20%) (3, 4). Three large first-line sorafenib studies also showed an incidence of hand-foot skin reaction ≥grade 3 of 0.4–1.3% in Western patients [3, 9, 24], which was lower than the corresponding rates in Asian patients (9.2–13.5%), including those in our study (9.2%).

Finally, there were some limitations of this study, including the potential selection bias associated with retrospective analyses, incomplete data collection, and heterogeneity in the clinicians' experiences of dose modification. Further investigations into the efficacy of sorafenib, as well as into its prognostic factors and toxicity profiles, are needed in Korea. It is crucial to establish new strategies to allow patients to receive continuous treatment without sacrificing either the efficacy or their quality of life.

## Conclusion

This study showed the efficacy and a tolerable safety of sorafenib in Korean mRCC patients as compared to that reported in other countries.

## Supporting Information

S1 Table An overall description of adverse events during sorafenib treatment was added in a supplementary file. (DOCX)
