# Supplementary material for: Efficacy and Safety of Sorafenib Therapy on Metastatic Renal Cell Carcinoma in Korean Patients: Results from a Retrospective Multicenter Study
Source: PLoS One. 2015 Aug 26;10(8):e0135165. doi: 10.1371/journal.pone.0135165 (PMC4550402; doi:10.1371/journal.pone.0135165)
Supplement: S1 Table — (DOCX) [file pone.0135165.s001.docx]

S1 Table. Overall adverse events during sorafenib treatment

|  | **First-line (N=116)** | | | | **Overall (N=177)** | | | |
| --- | --- | --- | --- | --- | --- | --- | --- | --- |
| **Adverse event** | **≥Grade 3** | | **Total** | | **≥Grade 3** | | **Total** | |
|  | **N** | **%** | **N** | **%** | **N** | **%** | **N** | **%** |
| **Constitutional symptoms** |  |  |  |  |  |  |  |  |
| Fatigue | 0 | 0.0 | 63 | 54.3 | 2 | 1.1 | 91 | 51.4 |
| Weight loss | 0 | 0.0 | 32 | 27.6 | 1 | 0.6 | 47 | 26.6 |
| **Gastrointestinal** |  |  |  |  |  |  |  |  |
| Diarrhea | 1 | 0.8 | 51 | 44.0 | 4 | 2.3 | 81 | 45.8 |
| Nausea | 0 | 0.0 | 27 | 23.3 | 0 | 0.0 | 41 | 23.2 |
| Vomiting | 1 | 0.8 | 25 | 21.6 | 1 | 0.6 | 34 | 19.2 |
| Anorexia | 3 | 2.5 | 50 | 43.1 | 4 | 2.3 | 69 | 39.0 |
| Heartburn | 1 | 0.8 | 4 | 3.4 | 1 | 0.6 | 5 | 2.8 |
| Taste alteration | 0 | 0.0 | 18 | 15.5 | 0 | 0.0 | 21 | 11.9 |
| Constipation | 0 | 0.0 | 14 | 12.1 | 0 | 0.0 | 20 | 11.3 |
| Dental problems | 1 | 0.8 | 4 | 3.4 | 1 | 0.6 | 10 | 5.6 |
| Hemorrhoids | 0 | 0.0 | 0 | 0.0 | 0 | 0.0 | 1 | 0.6 |
| Ileus | 0 | 0.0 | 4 | 3.4 | 0 | 0.0 | 5 | 2.8 |
| Ulcer, GI | 0 | 0.0 | 4 | 3.4 | 0 | 0.0 | 4 | 2.3 |
| Mucositis/stomatitis | 1 | 0.8 | 40 | 34.5 | 2 | 1.1 | 61 | 34.5 |
| **Dermatological** |  |  |  |  |  |  |  |  |
| Rash/desquamation | 3 | 2.5 | 49 | 42.2 | 4 | 2.3 | 56 | 31.6 |
| Rash: hand-foot skin reaction | 11 | 9.2 | 78 | 67.2 | 18 | 10.2 | 111 | 62.7 |
| Nail changes | 0 | 0.0 | 21 | 18.1 | 0 | 0.0 | 25 | 14.1 |
| Dermal change | 2 | 1.7 | 20 | 17.2 | 2 | 1.1 | 26 | 14.7 |
| Alopecia | 1 | 0.8 | 36 | 31.0 | 2 | 1.1 | 47 | 26.6 |
| **Cardiac** |  |  |  |  |  |  |  |  |
| Hypertension | 1 | 0.9 | 33 | 28.4 | 7 | 4.0 | 48 | 27.1 |
| Left ventricular diastolic dysfunction | 0 | 0.0 | 1 | 0.9 | 0 | 0.0 | 1 | 0.6 |
| Cardiac ischemia/infarction | 1 | 0.9 | 1 | 0.9 | 1 | 0.6 | 1 | 0.6 |
| Arrhythmia | 0 | 0.0 | 1 | 0.9 | 0 | 0.0 | 1 | 0.6 |
| **Hemorrhage/bleeding** |  |  |  |  |  |  |  |  |
| Hemorrhage, GI | 0 | 0.0 | 1 | 0.9 | 0 | 0.0 | 1 | 0.6 |
| Hemorrhage, GU | 0 | 0.0 | 3 | 2.6 | 0 | 0.0 | 3 | 1.7 |
| Hemorrhage, pulmonary | 0 | 0.0 | 1 | 0.9 | 0 | 0.0 | 1 | 0.6 |
| Hemorrhage, other | 0 | 0.0 | 2 | 1.7 | 0 | 0.0 | 3 | 1.7 |
| **Vascular,** thrombosis/embolism | 0 | 0.0 | 1 | 0.9 | 0 | 0.0 | 1 | 0.6 |
| **Infection** | 0 | 0.0 | 3 | 2.6 | 0 | 0.0 | 4 | 2.3 |
| **Endocrine,** hypothyroidism | 0 | 0.0 | 10 | 8.6 | 0 | 0.0 | 19 | 10.7 |
| **Hepatobiliary/pancreas,** cholecystitis | 0 | 0.0 | 1 | 0.9 | 0 | 0.0 | 2 | 1.1 |
| **Pulmonary/upper respiratory** |  |  |  |  |  |  |  |  |
| Dyspnea | 1 | 0.8 | 25 | 21.6 | 1 | 0.6 | 41 | 23.2 |
| Pleural effusion | 0 | 0.0 | 9 | 7.8 | 0 | 0.0 | 11 | 6.2 |
| Voice changes | 1 | 0.8 | 2 | 1.7 | 2 | 1.1 | 3 | 1.7 |
| **Other** |  |  |  |  |  |  |  |  |
| Edema | 0 | 0.0 | 11 | 9.5 | 0 | 0.0 | 15 | 8.5 |
| Pain | 2 | 1.7 | 24 | 20.7 | 3 | 1.7 | 45 | 25.4 |
| Wound complication, non-infectious | 0 | 0.0 | 2 | 1.7 | 0 | 0.0 | 2 | 1.1 |
| Other | 0 | 0.0 | 9 | 7.8 | 0 | 0.0 | 20 | 11.3 |
| **Hematologic** |  |  |  |  |  |  |  |  |
| Anemia | 8 | 6.9 | 49 | 42.2 | 10 | 5.6 | 77 | 43.5 |
| Leucopenia | 0 | 0.0 | 15 | 12.9 | 0 | 0.0 | 24 | 13.6 |
| Lymphopenia | 3 | 2.6 | 15 | 12.9 | 3 | 1.7 | 17 | 9.6 |
| Neutrophil/granulocyte (ANC/AGC) | 2 | 1.7 | 8 | 6.9 | 2 | 1.1 | 15 | 8.5 |
| Thrombocytopenia | 0 | 0.0 | 12 | 10.3 | 0 | 0.0 | 21 | 11.9 |
| **Non-hematologic** |  |  |  |  |  |  |  |  |
| Hypoalbuminemia | 0 | 0.0 | 23 | 19.8 | 0 | 0.0 | 35 | 19.8 |
| Elevated alkaline phosphatase | 0 | 0.0 | 15 | 12.9 | 0 | 0.0 | 32 | 18.1 |
| Elevated AST | 2 | 1.7 | 17 | 14.7 | 2 | 1.1 | 27 | 15.3 |
| Elevated ALT | 3 | 2.6 | 15 | 12.9 | 4 | 2.3 | 23 | 13.0 |
| Hyperbilirubinemia | 1 | 0.9 | 7 | 6.0 | 1 | 0.6 | 10 | 5.6 |
| Hypocalcemia | 1 | 0.9 | 17 | 14.7 | 2 | 1.1 | 39 | 22.0 |
| Hypercalcemia | 0 | 0.0 | 4 | 3.4 | 0 | 0.0 | 7 | 4.0 |
| Hypercholesterolemia | 0 | 0.0 | 9 | 7.8 | 0 | 0.0 | 12 | 6.8 |
| Elevated creatinine | 3 | 2.6 | 29 | 25.0 | 3 | 1.7 | 45 | 25.4 |
| Hyperglycemia | 0 | 0.0 | 19 | 16.4 | 3 | 1.7 | 46 | 26.0 |
| Elevated amylase | 2 | 1.7 | 18 | 15.5 | 5 | 2.8 | 24 | 13.6 |
| Elevated lipase | 4 | 3.4 | 17 | 14.7 | 8 | 4.5 | 30 | 16.9 |
| Hypomagnesemia | 0 | 0.0 | 2 | 1.7 | 0 | 0.0 | 2 | 1.1 |
| Hypophosphatemia | 9 | 7.8 | 20 | 17.2 | 14 | 7.9 | 34 | 19.2 |
| Hyperkalemia | 1 | 0.9 | 8 | 6.9 | 2 | 1.1 | 10 | 5.6 |
| Hypokalemia | 0 | 0.0 | 3 | 2.6 | 0 | 0.0 | 5 | 2.8 |
| Hyponatremia | 1 | 0.8 | 11 | 9.5 | 2 | 1.1 | 16 | 9.0 |
| Hypertriglyceridemia | 0 | 0.0 | 1 | 0.9 | 0 | 0.0 | 1 | 0.6 |
| Hyperuricemia | 0 | 0.0 | 17 | 14.7 | 1 | 0.6 | 31 | 17.5 |
| Proteinuria | 0 | 0.0 | 17 | 14.7 | 0 | 0.0 | 35 | 19.8 |
| **Total** | **71** | - | **1048** | - | **118** | **-** | **1595** | **-** |

GI, gastrointestinal; GU, gastric ulcer; ANC, absolute neutrophil count; AGC, absolute granulocyte count; AST, aspartate transaminase; ALT, alanine transaminase
